# Peripheral neuropathy in a family with Sandhoff disease and SH3TC2 deficiency

**DOI:** 10.1007/s00415-015-7683-x

**Published:** 2015-03-04

**Authors:** Christopher Grunseich, Alice B. Schindler, Ke-lian Chen, Dara Bakar, Ami Mankodi, Ryan Traslavina, Abhik Ray-Chaudhury, Tanya J. Lehky, Eva H. Baker, Nicholas J. Maragakis, Cynthia J. Tifft, Kenneth H. Fischbeck

**Affiliations:** 1 Neurogenetics Branch, National Institute of Neurological Disorders and Stroke, NIH, Bethesda, MD 20892 USA; 2 Surgical Neurology Branch, National Institutes of Neurological Disorders and Stroke, NIH, Bethesda, MD 20892 USA; 3 Electromyography Section, National Institute of Neurological Disorders and Stroke NIH, Bethesda, MD 20892 USA; 4 Department of Radiology and Imaging Sciences, Clinical Center, NIH, Bethesda, MD 20892 USA; 5 Department of Neurology, Johns Hopkins University School of Medicine, Baltimore, MD 21205 USA; 6 Office of the Clinical Director, National Human Genome Research Institute, NIH, Bethesda, MD 20892 USA

Dear Sirs,

Sandhoff disease is a lysosomal storage disease caused by a deficiency of beta-hexosaminidase. Affected individuals present with a wide spectrum of clinical manifestations, ranging from psychomotor impairment and death in the infantile form to motor neuron disease and autonomic dysfunction in the adult form [1, 2]. Here, we present a family with predominantly sensory neuropathy that was found to have both Sandhoff disease and deficiency of SH3TC2.

**Fig. 1:**
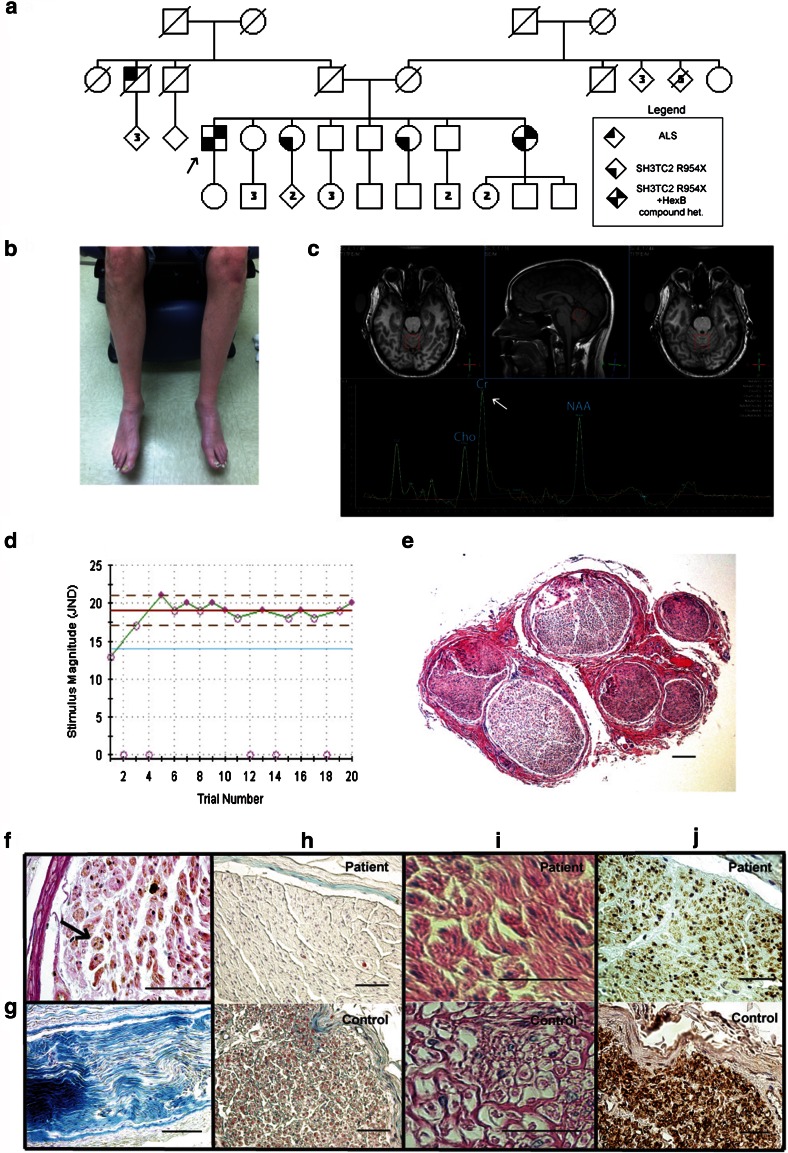
Family pedigree and phenotypical evaluations in the proband. **a** Family pedigree. The *arrow* indicates the proband. Three of the proband’s sisters were also heterozygous for the *SH3TC2* mutation, but only the two affected individuals had compound heterozygous mutations in *HEXB* and the *SH3TC2* mutation. A paternal uncle was diagnosed with amyotrophic lateral sclerosis (ALS). **b** A photo of the proband showing atrophy and bluish discoloration of the feet. **c** MR spectroscopy (MRS) of the superior cerebellar vermis showing low *N*-acetylaspartate (NAA)/creatine ratio, low choline (Cho)/creatine (Cr) ratio, and normal choline/NAA ratio (the *arrow* indicates the elevated creatine peak). MRS of the same location in the proband’s affected sister showed a low choline/creatine ratio. Normal reference ranges were established from five controls. **d** The vibratory detection threshold (VDT) for the hand, showing increased detection threshold stimulus magnitude just noticeable difference (JND). *Shaded circles* indicate when a stimulus was detected. No stimulus was administered in trials 2, 4, 12, 14, and 18. The average threshold observed is 19.0 JND (*red line*), higher than the normative control average of 14.4 JND (*blue line*). **e** Hematoxylin and eosin (H+E) staining of the proband’s sural nerve demonstrating a reduction of large myelinated nerve fibers; *bar* 100 μm. **f** Staining with periodic acid–Schiff (PAS) (*pink*) and SH3TC2 (*brown*) in Schwann cells. *Arrow* indicates miniature onion bulb with multiple Schwann cell nuclei. **g** Luxol fast staining of a longitudinal section showing evidence of demyelination. **h** Gomori trichrome staining showing a reduction in myelin staining (*red*) in the patient as compared to control. **i** PAS staining in patient’s Schwann cells showing positive staining compared to control. **j** Staining with an antibody for SH3TC2 shows a reduction in staining in the patient compared to control. *Bar* 50 μm for (**f**–**j**)

A 46-year-old man had weakness, atrophy, and paresthesia with loss of sensation in his hands and feet for 30 years (Fig. 1a). He also had severe diarrhea several times per week, decreased sweating over his feet, and episodes of lightheadedness. On examination, he was found to have marked upper and lower extremity weakness and muscle atrophy distally, with the legs more severely affected (Fig. 1b). Although hyperreflexia was found in the upper extremities, reflexes in the lower extremities were reduced. Electromyography showed evidence of chronic neurogenic changes, and nerve conduction studies showed normal motor conduction velocities (patient 40–67 m/s, sibling 39–58 m/s). However, sensory nerve conduction studies were non-responsive in the proband and either non-responsive or reduced in amplitude and velocity (41 m/s) in the sibling. Autonomic testing (QSWEAT, Heart Rate Response to Deep Breathing, Valsalva, Tilt Table) and quantitative sensory testing (Vibratory Detection Threshold and Cooling Detection Threshold) showed evidence of autonomic dysfunction consistent with a severe small unmyelinated fiber and large myelinated fiber sensory neuropathy (Fig. 1d) [3]. Laboratory testing for acquired causes of neuropathy was negative. An evaluation of the proband’s 57-year-old sister showed a similar pattern of sensory, motor, and autonomic involvement, with symptom onset in her 20s. Both individuals did not show evidence of macular abnormalities; however, mild splenomegaly was found in the sister (span 14.2 cm).

After negative genetic testing (Athena Diagnostics Inc.) of ten candidate neuropathy genes (*PMP22, Cx32, MPZ, PMP22, EGR2, NFL, PRX, GDAP1, LITAF, MFN2*), whole exome sequencing (NISC/NHGRI/NIH) of the proband identified a common 1250C>T mutation (P417L) in *HEXB* and a previously reported [4] 2860C>T mutation (R954X) in *SH3TC2*, which was confirmed by Sanger sequencing (GeneDx). Further testing through multiplex ligation-dependent probe amplification (MLPA, GeneDx) showed that the proband is also heterozygous for a 16-kb deletion that includes exons 1–5 of the *HEXB* gene. The three variants were also confirmed in the affected sister, and although two of the unaffected sisters were found to have the *SH3TC2* mutation, neither have both *HEXB* mutations. MR spectroscopy of the superior cerebellar vermis detected elevated creatine in the proband (Fig. 1c). Serum testing in both affected siblings showed low total hexosaminidase activity of 1.4 and 1.0 nmol/min/mL (normal 10.4–23.8) and an increased fraction of HEXA activity at 91 and 100 % (normal 56–80 %). A sural nerve biopsy from the proband showed evidence of a severe mixed axonal and demyelinating neuropathy (Fig. 1e–i) and a reduction in SH3TC2 immunoreactivity (Fig. 1j).

The adult form of beta-hexosaminidase deficiency is characterized by upper and lower motor neuron dysfunction, dysautonomia, and ataxia. The amino acid change in HEXB at position 417 is a common gene mutation in adult Sandhoff disease patients [5]. The early and severe presentation of sensory findings in both of these siblings is not a typical presentation of the disease [6–8]. The nonsense mutation observed in this family is predicted to reduce levels of SH3TC2, consistent with the pattern detected on peripheral nerve staining.

Given the unusual presentation with pronounced sensory neuropathy in this HEXB family and the evidence of myelin defects on nerve biopsy, not explained by Sandhoff disease alone, it is plausible that the deficiency in SH3TC2 may be modifying the HEXB phenotype. SH3TC2 is localized to the endosome, and disease-causing missense mutations mistarget the protein away from this location and result in a demyelinating neuropathy [9]. It is possible that the accumulation of GM2 ganglioside in HEXB-deficient endosomes and lysosomes may be preferentially damaging to tissues that also have a deficiency of SH3TC2.
